# Measuring social capital of hospital management boards in European hospitals: A validation study on psychometric properties of a questionnaire for Chief Executive Officers

**DOI:** 10.1186/s12913-021-07067-y

**Published:** 2021-10-01

**Authors:** Antje Hammer, Onyebuchi A. Arah, Russell Mannion, Oliver Groene, Rosa Sunol, Holger Pfaff, Kyung-Eun Choi

**Affiliations:** 1 Institute of Medical Sociology, Health Services Research, and Rehabilitation Science, Faculty of Medicine and University Hospital Cologne, Faculty of Human Sciences, University of Cologne, Cologne, Germany; 2 Department of Epidemiology, UCLA Fielding School of Public Health, Los Angeles, USA; 3 UCLA Center for Health Policy Research, Los Angeles, USA; 4 Health Services Management Centre, School of Social Policy, University of Birmingham, Birmingham, United Kingdom; 5 OptiMedis AG, Hamburg, Germany; 6 Avedis Donabedian Research Institute (FAD), Barcelona, Spain; 7 Universitat Autònoma de Barcelona, Barcelona, Spain; 8 Red de investigación en servicios de salud en enfermedades crónicas (REDISSEC), Donostia, Spain; 9 Center for Health Services Research, Brandenburg Medical School Theodor Fontane, Rüdersdorf b. Berlin/Neuruppin, Germany

**Keywords:** Social capital, validation, psychometric properties, hospital management, hospital executives, leadership, work environment, organization

## Abstract

**Background:**

The commitment of hospital managers plays a key role in decisions regarding investments in quality improvement (QI) and the implementation of quality improvement systems (QIS). With regard to the concept of social capital, successful cooperation and coordination among hospital management board members is strongly influenced by commonly shared values and mutual trust. The purpose of this study is to investigate the reliability and validity of a survey scale designed to assess *Social Capital* within hospital management boards (SOCAPO-B) in European hospitals.

**Methods:**

Data were collected as part of the EU funded mixed-method project “Deepening our understanding of quality improvement in Europe (DUQuE)” from 210 hospitals in 7 European countries (France, Poland, Czech Republic, Germany, Portugal, Spain, and Turkey). The Chief Executive Officers (CEOs) completed the SOCAPO-B scale (six-item survey, numeric scale, 1=‘strongly disagree’ to 4=‘strongly agree’) regarding their perceptions of social capital within the hospital management board. We investigated the factor structure of the social capital scale using exploratory and confirmatory factor analyses. Internal consistency was assessed using Cronbach’s alpha, while construct validity was assessed through Pearson’s correlation coefficients between the scale items.

**Results:**

A total of 188 hospitals participated in the DUQuE-study. Of these, 177 CEOs completed the questionnaire(172 observations for social capital) Hospital CEOs perceive relatively high social capital among hospital management boards (average SOCAPO-B mean of 3.2, SD = 0.61). The exploratory factor analysis resulted in a 1-factor-model with Cronbach’s alpha of 0.91. Pearson’s correlation coefficients between the single scale items ranged from 0.48 to 0.68.

**Conclusions:**

The SOCAPO-B_−_scale can be used to obtain reliable and valid measurements of social capital in European hospital management boards, at least from the CEO’s point of view. The brevity of the scale enables it to be a cost-effective and tool for measuring social capital in hospital management boards.

**Trial registration:**

This validation study was not registered.

## Background

Hospital quality management is still in its infancy but developing rapidly in response to new pressures and demands. Modern hospitals around the globe are faced with a range of complex challenges (financial, technological and population based). Improving the quality of care in such circumstances has become a critical issue for hospitals to grapple with around the globe. Hospitals in Europe have adapted their services to meet these challenges and are implementing a range of quality improvement (QI) strategies and attempt to improve the quality and safety of patient care including incident reporting systems, implementation of evidence-based guidelines, breakthrough projects, audits, and a variety of performance indicators and metrics. There are also increasing external pressure on hospitals to provide better, safer care to patients. As such, an increased importance of establishing quality improvements systems (QIS) within health care organizations has become a vital part of QI-strategies in hospitals. Nevertheless, the application of newly developed operational standards and guidelines, information and scientific results appears to advance only slowly and unevenly both within and across countries [1].

Investments in patient safety and the implementation of QIS are to a large extent based on the decisions of senior managers [2, 3]. Moreover, managers sitting at the apex of the organisation are responsible for setting strategic direction, crafting strategy and creating organizational cultures which support (or at least do not hinder QI-efforts [4]. Therefore, the quality of hospital managers leadership and decision-making is an important organizational capability for successfully implementing QI [4–6]. In highly fragmented systems such as health care organizations, achieving effective relationships based on trust is a major challenge. From the perspective of an individual hospital, such relationship building and performing has to be nurtured in the context of interprofessional teamworking and multi-hierarchical collaboration. Organization research has highlighted that supervision, standardization and mutual adjustment (informal communication and the ability to adapt to each other) are key mechanisms for a productive organisational culture [7]. Effective organizational relationship building is positively associated with better quality of care [8] and hospital profitability [9].

*Social capital* is a specific form of organizational resource. The French sociologist Pierre Bourdieu conceives of social capital as network of relationships that allow access to resources. The amount of social capital is dependent on (1) grade of expansion regarding the social network, and (2) the volume of capital that can be accessed via the network [10]. According to Putnam [11], a deficit of social capital leads to inefficiency and hampers coordination and cooperation for mutual benefit [11]. A specific characteristic of social capital is that, in contrast to other forms of capital, the resource does not lie in the social actors themselves (such as human capital) or the physical means of production (physical capital), but rather in the structure of relationships [12]. According to Coleman closeness and trustworthiness of social structure are two essential prerequisites for enhancing social capital. That is, that when members of a collective perceive close relationships, they are more likely to help each other, to create effective procedures and share key information [12]. In Germany, the construct of social capital has been used, in a variety of forms for over 20 years to assess the quality and structure of relationships in organisations [13]. In healthcare organizations, social capital has a special significance being associated as it is with higher job satisfaction among clinicians [14], reduced ‘burnout’ among employees [15, 16], improved risk management [17] and better coordination [8]. Organizations with high levels of social capital are characterized by social relations between the members of an organization based on mutual trust and understanding as well as shared convictions and values [18]. Such cultures influence collective organisational endeavours and supports a specific understanding of patient safety and quality of care.

The quality of leadership depends on commonly shared values and mutual trust among hospital management board members. From a social capital perspective, these are essential requirements for successful cooperation and coordination within groups – including the hospital management board [19]. Although it has been used in several earlier studies, the social capital scale has not been intensively validated. An earlier study on employees’ social capital (SOCAPO-E) has recently been published [20]. In this study we focus on CEOs. The development of a brief scale consisting of six items was based on sociological principles relating to social capital described by Bourdieu [10], Coleman [21], Putnam [11, 22], and Fukuyama [23] and especially the concept of community [24]. The general social items of the SOCAPO-E [20] adapted to hospital management boards to measure the social capital within the board from the perspective of board members (SOCAPO-B). We incorporated this scale into the CEO’s questionnaire asking them about their perceptions regarding levels of social capital within the hospital (management) board (SOCAPO-B). Therefore, this study set out to investigate the reliability and validity of a survey scale to assess *Social Capital* within hospital management boards (SOCAPO-B) in hospitals in 7 European countries.

## Methods

### Setting, study design and population

This paper is based on data from the parent project “Deepening our understanding of QI-in Europe (DUQuE)” funded by the EU 7th Research Framework Program. More details on the study setting, population, and design have previously been published [25, 26]. DUQuE sought to study the effectiveness of QIS in European hospitals. The study used a multi-method approach to data collection and measurement. Overall, we approached 210 randomly selected hospitals in 7 countries (France, Poland, Germany, Czech Republic, Portugal, Spain, and Turkey). The sample was restricted to hospitals with more than 130 beds that provide for acute myocardial infection, stroke, hip fracture, and delivery. Data were collected at four levels (hospital level, departmental level, professional level, and patient level). However, the analyses presented in this article consists solely of hospital-level constructs measured within a survey for Chief Executive Officers (CEOs). Those were selected as key informants, since the CEOs are considered to have referent and informational power in the hospital and able to implement new structures, strategies and incentives to improve hospital performance. Data were collected through a web-based questionnaire between May 2011 and February 2012.

### Measure: social capital of hospital management boards (SOCAPO-B)

The variable ‘social capital’ of management boards’ (SOCAPO-B) was devised to measure two key features of the construct 1) common values and 2) perceived mutual trust in organizations [18]. Originally, the six-item scale was developed to measure communal social capital of healthcare organizations out of the perspectives of employees (SOCAPO-E) [27]. The variable consisted of six items and has been used in several previous studies [5, 28–31]. The reliability of the social capital scale - tested in a prior German study - is high with a Cronbach’s alpha of 0.83 [27]. Answers were scaled on a 4-point Likert scale (range from 1=‘strongly disagree’ to 4=‘strongly agree’). Higher values indicate that CEOs have a higher perception of social capital in the hospital management board. The six questions of the scale are: When thinking about your Hospital (Management) Board, how much do you agree with the following statements? (1 = strongly disagree, 2 = somewhat disagree, 3 = somewhat agree, 4 = strongly agree): Within our Hospital (Management) Board… (1) … there is unity and agreement, (2) … we trust one another, (3) … there is a “we feeling” among Board members, (4) … the work climate is good, (5) … the willingness to help one is great, (6) … we share many common values.

### Translation and adaptation

To ensure comparability and interpretability of data in the cross-national contexts and to obtain practical implications, rigorous study standards regarding study design, translation processes, administration and country coordination must be upheld. In accordance with the recommendations of Guillemin et al. [32] single items from the original social capital scale were translated into English by three independent professional translators/native speakers. The following backwards translations into German were undertaken by another three professional translators/native speakers. Backwards translation has been independently reviewed and assessed by a team of researchers from the Institute of Medical Sociology, Health Services Research, and Rehabilitation Science. Based on this review, the most appropriate translations were selected. In the second step - again using a forward-backward method [32] - the SOCAPO-B-scale was translated together with all DUQuE-data collection tools and as part of the CEO’s questionnaire by the Country Coordinators – who were responsible for data collection in each country. Differences in language, cultures, contextual factors and differences in organisations settings within the countries have been taken into consideration [33, 34].

### Statistical analyses

Before embarking on the in-depth analysis, respondents with missing values of > 30 % in social capital items were excluded because of poor data quality. The final dataset used in this analysis contains only hospitals with complete records on all six social capital items.

First, we used descriptive statistics to describe the sample data used for this analysis. For categorical variables we calculated frequencies and percentages. For continuous variables, we calculated the minimum, maximum, mean and standard deviation. For sampling adequacy, Kaiser-Meyer-Olkin (KMO) and Measure of Sample Adequacy (MSA) coefficients have been calculated. Values of > 0.60 indicate a good applicability and values of > 0.90 would indicate a perfect applicability [35].

Prior to undertaking factor analyses, we calculated Pearson’s correlation coefficients for all 6 social capital items (inter-item correlation) and between items and SOCAPO-B-scale (item-total correlation). According to Campbell and Fiske [36], we determined a cut-off value of ≥ 0.70 for inter-item correlation. Higher values indicate that the items measure the same concepts, while values < 0.30 indicate a poor relationship between the single items [35]. Additionally, we examined the homogeneity of SOCAPO-B-scale using item-total correlations. Coefficients of ≥ 0.40 would indicate adequate evidence of scale homogeneity. This analysis was followed by a principal component analysis (PCA) in order to study the component structure of the SOCAPO-B-scale. We set eigenvalues above 1.0 and used Varimax rotation to optimize factor structure [35]. Cut-off value for factor loadings was set to 0.4 to minimize item cross-loadings [35].

During the next stage we performed a confirmatory factor analysis (CFA) [37] to test whether the original developed factor structure of the SOCAPO-B-scale fitted our data. In accordance with Kline [37] and Hair et al. [38] we used global and local fit indices for assessing the appropriateness of the model. Goodness-of-fit was assessed with the Chi-squared values indicating differences in observed and expected covariance matrices [37, 38]. Since Chi-squared value is sensitive to the sample size, we calculated the normed Chi-squared value (Chi²/df) setting a cut-off value of ≤ 2.5 [39]. We also computed the Goodness of Fit Index (GFI) as absolute model fit measure with recommended cut off value of > 0.90 [38]. Additionally, we assessed several incremental and descriptive measures of model fit: (1) Comparative Fit Index (CFI); (2) Bentler-Bonett Normed Fit Index (NFI); (3) Bentler-Bonett Non-normed Index (NNFI); (4) Adjusted Goodness od Fit Index (AGFI); (5) and Root Mean Square Error of Approximation (RMSEA). Because the Standardised Root Mean Residual (SRMR) might be biased with less than 12 observed variables [38], we refrained from calculating this measure. With a sample size of N less than 250 and the number of observed variables less than 12, cut-off values were determined as follows: CFI, NNFI and AGFI ≥ 0.90, NFI ≥ 0.95, RMSEA (with CFA ≥ 0.90) ≤ 0.07 [38–40]. In order to assess the degree to which the scale is reliable and valid we used local fit indices with the following criteria and cut-off values: Indicator reliability ≥ 0.30, factor reliability ≥ 0.60 and Average Variance Extracted (AVE) ≥ 0.50 [41].

Finally, the internal consistency (reliability) was examined through Cronbach’s alpha, which is a coefficient used to assess the interrelatedness of the items and the homogeneity of the scale”, and in addition underline the meaning of the values 0.70 and 0.90 that you have indicated [35, 37].

All analyses were conducted using SAS version 9.3 (SAS Institute Inc. Cary, NC, USA).

## Results

### Descriptive statistics

Overall, 188 out of 210 hospitals (response rate = 89.5 %) participated in the DUQuE-study. Of these, we received 177 completed questionnaires from hospital CEOs. Due to missing values, we count 172 observations for social capital. The final dataset used for these analyses contains only hospitals with complete records on all SOCAPO-B-items (*N* = 172).

Characteristics of the hospitals used in this analysis are presented in Table 1. Almost 81.4 % (*N* = 140) of the hospitals were public hospitals, while 18.6 % (*N* = 32) were non-public hospitals. The sample included 42.4 % (*N* = 73) teaching hospitals and 57.6 % (*N* = 99) non-teaching hospitals. 18 hospitals (10.5 %) had < 200 beds, 74 hospitals (43.0 %) had 200–500 beds, 54 hospitals (31.4 %) had 501–1000 beds and 26 hospitals (15.1 %) had > 1000 beds.

**Table.1:** Characteristics of hospitals used in analysis (*N* = 172)

	N	%
**Country code**		
Czech Republic	29	16.9
France	23	13.4
Germany	12	6.98
Poland	27	15.7
Portugal	28	16.3
Spain	27	15.7
Turkey	26	15.1
**Public Hospital**		
private	32	18.6
public	140	81.4
**Teaching Hospital**		
non-teaching	99	57.6
teaching	73	42.4
**Approximate number of beds in hospital**		
< 200	18	10.5
200–500	74	43.0
501–1000	54	31.4
> 1000	26	15.1

The average mean of the SOCAPO-B-scale is 3.25 (SD = 0.61). The single items scores range between 3.10 (for board1) and 3.36 (for board4). The average mean of social capital per country (results not presented in a table) range from 3.02 (SD = 0.59) and 3.41 (SD = 0.61) (Table 1). The mean values for social capital (1 = ‘I strongly disagree’ and 4 = ‘I strongly agree’) show that hospital’s CEOs perceive a relatively high social capital within the hospital management board. Descriptive statistics, including means and standard deviations for all SOCAPO-B-items and the SOCAPO-B-scale are presented in Table 2.

**Table.2:** Descriptive SOCAPO-B-scale and item (*N* = 172)

	Mean	Min	Max	Std	Var
**Social capital (Hospital level)**	3.25	1.33	4.00	0.605	0.366
board1: unity and agreement	3.10	1.00	4.00	0.750	0.562
board2: trust	3.28	1.00	4.00	0.746	0.556
board3: we feeling	3.33	1.00	4.00	0.741	0.548
board4: good work climate	3.36	1.00	4.00	0.674	0.454
board5: willingness to help each other	3.17	1.00	4.00	0.726	0.527
board6: share common values	3.23	1.00	4.00	0.760	0.577
**Social capital (Hospital level) per Country**					
Czech Republic	3.08	1.50	4.00	0.592	0.350
France	3.33	2.50	4.00	0.516	0.266
Germany	3.13	1.67	4.00	0.686	0.470
Poland	3.03	1.50	4.00	0.593	0.352
Portugal	3.41	1.67	4.00	0.674	0.454
Spain	3.31	1.33	4.00	0.595	0.354
Turkey	3.40	2.17	4.00	0.538	0.289

KMO coefficient value is 0.91, which indicates that the patterns of correlations are very compact and that a factor analysis is therefore appropriate for our data [35]. All SOCAPO-B-items reached superior MSA coefficients values of > 0.90 (range between 0.90 (board1) and 0.92 (board3)). Therefore, both the KMO test and the MSA test indicate that the data appropriately fit the criteria for factor analysis [35].

### Factor analysis

Results on Inter-item and inter-total correlations are in Table 3. Inter-item correlation range between 0.48 (board1 and board6) and 0.68 (board2 and board4). Thus, all correlations reached acceptable values between 0.20 and 0.70. Item-total correlations range from 0.80 to 0.86 indicating sufficient scale homogeneity.

**Table.3:** Inter-item and inter-total correlations

	board1	board2	board3	board4	board5	board6
Social capital (Hospital level)	0.80	0.85	0.83	0.86	0.83	0.80
board1: unity and agreement	1.000	0.66	0.60	0.64	0.56	0.48
board2: trust		1.000	0.65	0.70	0.61	0.62
board3: we feeling			1.000	0.67	0.64	0.58
board4: good work climate				1.000	0.66	0.64
board5: willingness to help each other					1.000	0.63
board6: share common values						1.000

Note: All correlations are significant at p ≤ 0.001

PCA resulted in 1 component with eigenvalue greater 1.0 supporting the assumption of a single factor structure for SOCAPO-B-scale. This one factor explains a total variance of 68.45. Rotating factor structure was not required. However, items load high on the extracted component with a range between 0.79 and 0.87 (Table 5).

Results from CFA indicated that the assumed factor structure fits the data for DUQuE’s CEOs. The factor model exhibited an acceptable-to-good global data fit (Table 4). Furthermore, the local fit indices were considered acceptable (Table 5). Regarding the indicator reliability all items exceeded the acceptable values. Moreover, the SOCAPO-B-scale reached the recommended critical values for the factor reliability and reached an adequate AVE value. These results suggested good convergent validity.

Overall Cronbach’s alpha reached a value of 0.91, above the recommended cut-off of 0.90 suggesting a close relation between single items. Dropping any single items would lead to an increase in Cronbach’s alpha.

**Table.4:** Model fits for SOCAPO-B-scale

Model Fit Index	Criterion (N < 250 and m < 12)	Fit index DUQuE sample
Chi²		12.297
df		9
*p*	Not significant p expected	0.197
Chi²/df	< 2.5	1.366
CFI	≥ 0.97	0.995
NFI	≥ 0.95	0.980
NNFI	≥ 0.97	0.991
GFI	> 0.90	0.977
AGFI	> 0.90	0.946
RMSEA	< 0.08 (with CFI ≥ 0.97)	0.046

*NOTE*: For thresholds of acceptable fit see Hair et al. [29], Hu and Bentler [31] and Bollen [30]

**Table.5:** Local fit, factor loadings and alpha if item deleted of SOCAPO-B-items

Item	Indicator reliability	Factor reliability	AVE	Factor loading component matrix	Alpha if item deleted
board1: unity and agreement	0.56	0.91	0.62	0.79	0.90
board2: trust	0.67			0.85	0.89
board3: we feeling	0.63			0.83	0.89
board4: good work climate	0.71			0.87	0.88
board5: willingness to help each other	0.62			0.83	0.89
board6: share common values	0.55			0.79	0.90

## Discussion

The aim of the study was to examine the psychometric properties of a theory-driven questionnaire measuring social capital among senior managers / hospital management boards in hospitals. The tests of the construct validity indicate that the SOCAPO-B-scale is a valid and reliable method of measuring social capital in European hospital management boards from the CEO’s point of view. Except for Cronbach’s Alpha exceeding minimal the suggested cut-off value of 0.90, results showed an acceptable level of reliability for SOCAPO-B. Based on the average mean of the SOCAPO-B-scale of 3.25 (SD = 0.61), we conclude that hospital’s CEOs perceive a relatively high social capital within the hospital management board.

Although the scale has been used in Germany for over 20 years, only a recent study published its psychometric properties based on employees’ data (SOCAPO-E) embedded in a German survey [20]. Our data were collected as part of the large scale European DUQuE-study on the effectiveness of QIS of hospitals. We believe this to be the first comprehensive study of QIS with an *a priori* development of a coherent theory to direct data collection and analysis, the variety of 7 European countries and the amount of standardized data [42]. Another major strength of the study is the high response rates of 89.5 %. Asking key informants may improve the understanding of organizational processes and management decisions made. They provide aggregated organizational data by reporting organizational and group properties rather than personal attitudes and behavior [43, 44]. A major limitation is the cross-sectional nature of the study design which does not allow for causal conclusions nor conclusions about the sensitivity of change in the scale. Repeated measurements could have further supported the validity of the scale. Furthermore, we cannot be sure that the SOCAPO-B-scale structure is stable in all countries. Especially, the unequally distributed numbers of participating hospitals per country, may have influenced the results. We believe that the general rationale of the validation is independent of country effects and hope that future studies will show to what point the scale is also applicable in other regions. Psychometric testing in future assessments in similar settings would also help to confirm the results. Within the scope of this study, we were not able to examine any relationships between social capital and hospital management outcomes, but this has been conducted and published elsewhere [45]. That the social capital scale was embedded in a larger questionnaire within the DUQuE-study could have influenced the responses of the CEO’s. However, we assumed that the length of the questionnaire would not necessarily affect the validity or reliability of the SOCAPO-B-scale. There is an ongoing discussion, whether scale with even or odd numbers of options should be favored. Particularly for new constructs, to yield a forced tendency may be of advantage, since a middle category may be used as “I do not know” category with ambiguity about the respondent meant indifference or being torn between the different dimensions. Unmotivated respondents may reduce their cognitive effort by selecting the middle category as a consequence of satisficing [46]. Nonetheless, further analyses on validity and reliability should be performed using the SOCAPO-B questionnaire only. A further limitation is that questioning key informants can result in inaccurate or biased data, because informants are either motivated or not to do so [47]. Also, the views of CEOs may vary from front line staff or mid-tier executives lower down the organizational hierarchy. Future studies could explore these possible differences.

A small number of studies already suggest a vital role of social capital as a resource in health care organizations [28, 45]. Relevant processes encompass quality and risk management or strengthening patient-physician relationships. The fact that social capital is associated with work engagement, well-being and depressive symptoms among staff underlines its significance for general health and overall performance in health care organizations [20]. Recent social and economic changes in analyzed countries may be considered in future studies. Although social climate is an important asset in hospital quality development, it has not received much attention, in the literature. The brevity of the scale allows to include this important aspect in wider surveys on other areas of health services research (patient safety, general quality improvement, employees’ satisfaction, patients’ satisfaction).

## Strengths and limitations of this study

From the management perspective, it is necessary to assess how the senior management of a hospital (especially the CEOs), assesses social capital within the management board, as they provide aggregated organizational data by reporting organizational and group properties rather than personal attitudes and behavior. We believe this to be the first comprehensive study of QIS using an *a priori* development of a coherent theory to direct data collection and analysis, the variety of 7 European countries and the amount of standardized data with high response rates of 89.5 %. Based on the positive results regarding the construct validity and an acceptable reliability for the social capital scale within the hospital management board, we recommend the use of this tool in future research. A major limitation is the cross-sectional nature of the study design which does not allow for conclusions about causality or the sensitivity of change in the scale.

## Conclusions

Decisions regarding investments in QI-and the implementation of QIS are based largely on the commitment of hospital management. Managers set the strategic direction of the organization, craft organisational strategy and guide efforts towards successful quality improvement. Mutual trust and shared values substantially influence the quality of cooperation, communication and ability to act as an organizational group. Therefore, we believe that the assessment of commonly shared values and mutual trust can support the implementation of QIS. Based on our findings regarding validity and reliability, we can recommend the SOCAPO-B-scale for the future assessment of social capital in hospital management boards. Finally, future studies in this area would benefit from examining social capital lower down the hierarchy, at the departmental, ward and team level.

## Data Availability

The datasets used and/or analyzed during the current study are available from the study group on reasonable request. Please contact the corresponding author.

## Abbreviations

AGFI: Adjusted Goodness od Fit Index
AVE: Average Variance Extracted
CEO: chief executive officer
CFA: confirmatory factor analysis
CFI: Comparative Fit Index
DUQuE: “Deepening our understanding of quality improvement in Europe”
GFI: Goodness of Fit Index
KMO: Kaiser-Meyer-Olkin
MSA: Measure of Sample Adequacy
NFI: Normed Fit Index
NNFI: Non-normed Index
QI: Quality improvement
QIS: Quality improvement systems
SOCAPO-B: social capital within hospital management boards
SOCAPO-E: social capital of healthcare organizations out of the perspectives of employees
SRMR: Standardised Root Mean Residual
RMSEA: Root Mean Square Error of Approximation
